# Unveiling the Dementia Burden: A National Prevalence Study in Morocco

**DOI:** 10.1002/alz70860_106906

**Published:** 2025-12-23

**Authors:** Abderrahmane CHAHIDI, Zouhayr SOUIRTI, Najat Belarbi, Said Boujraf

**Affiliations:** ^1^ SIDI MOHAMMED BEN ABDELLAH UNIVERSITY, Faculty of Psychology, Fez, FEZ, Morocco; ^2^ Clinical Neurosciences Laboratory, University Medical School, Sidi Mohammed Ben Abdellah University, FEZ, Morocco; ^3^ Moroccan Society of Clinical Neurophysiology, Marrakech, Morocco; ^4^ Clinical Neurosciences Research Laboratory, University Medical School, Sidi Mohammed Ibn Abdellah University, Fez, FEZ, Morocco; ^5^ Department of Neurology, Hassan II University hospital, Fez, Morocco; ^6^ Sidi Mohammed Ibn Abdellah University, Department of Psychology, Fez, Morocco; ^7^ Clinical Neurosciences Laboratory, University Medical School, Sidi Mohammed Ben Abdellah University, Fez, Morocco

## Abstract

**Background:**

Dementia is a growing global health crisis, affecting over 55 million people, with a disproportionate burden in low‐ and middle‐income countries. Its prevalence is projected to triple by 2050, primarily due to aging populations. Despite this, Morocco lacks comprehensive epidemiological data on dementia. This study aims to bridge this gap by estimating the national prevalence of dementia and its subtypes, particularly Alzheimer's disease and mild cognitive impairment (MCI).

**OBJECTIVES:**

✔ Estimate the prevalence of dementia among Moroccan adults.

✔ Assess the burden of Alzheimer's disease and other neurodegenerative causes.

✔ Examine MCI as a precursor to dementia.

✔ Identify regional and demographic variations.

✔ Provide data to guide national dementia policies.

**Method:**

Study Design: Large‐scale cross‐sectional epidemiological study.

Sampling: Nationally representative sample (50+ years) from urban and rural areas.

Data Collection: Telephone survey using a validated cognitive screening questionnaire.

Screening: T‐MoCA test (Moroccan dialect adaptation).

Confirmation: Neurologists conduct in‐depth assessments (DSM‐5 & WHO dementia criteria).

Hospital‐based validation: Neuroimaging & biomarker analysis for suspected Alzheimer's cases.

Statistical Analysis: Descriptive and multivariate logistic regression models.

Data analysis using R software (version 3.6.1).

**Result:**

Dementia burden is rising, with notable urban‐rural disparities.

Alzheimer's disease is the leading cause, followed by vascular dementia.

Key risk factors:

✔ Low education strongly correlates with cognitive decline.

✔ Cardiovascular diseases (hypertension, diabetes, stroke) increase dementia risk.

✔ Women are disproportionately affected, both as patients and caregivers.

✔ Limited awareness and delayed diagnosis hinder early intervention.

**Conclusion:**

This first national dementia prevalence study underscores urgent policy needs.

✔ Early detection programs for timely diagnosis.

✔ Public health interventions targeting modifiable risk factors.

✔ Caregiver support initiatives to mitigate social and economic impact.

✔ National dementia prevention strategies aligned with WHO recommendations.

Future Directions: Expansion of longitudinal studies, genetic research, and intervention trials for Morocco's aging population.

